# Design of a Porous Silicon Biosensor: Characterization, Modeling, and Application to the Indirect Detection of Bacteria

**DOI:** 10.3390/bios14020104

**Published:** 2024-02-17

**Authors:** Roselien Vercauteren, Clémentine Gevers, Jacques Mahillon, Laurent A. Francis

**Affiliations:** 1 Institute of Information and Communication Technologies, Electronics and Applied Mathematics, Université Catholique de Louvain, 1348 Louvain-la-Neuve, Belgium; roselien.vercauteren@vocsens.com (R.V.); clementine.gevers@uclouvain.be (C.G.); 2 Laboratory of Food and Environmental Microbiology, Earth and Life Institute, Université Catholique de Louvain, 1348 Louvain-la-Neuve, Belgium; jacques.mahillon@uclouvain.be

**Keywords:** biosensor, design, porous silicon, membranes, bacterial detection, endolysins

## Abstract

The design of a porous silicon (PSi) biosensor is not often documented, but is of the upmost importance to optimize its performance. In this work, the motivation behind the design choices of a PSi-based optical biosensor for the indirect detection of bacteria via their lysis is detailed. The transducer, based on a PSi membrane, was characterized and models were built to simulate the analyte diffusion, depending on the porous nanostructures, and to optimize the optical properties. Once all performances and properties were analyzed and optimized, a theoretical response was calculated. The theoretical limit of detection was computed as 10^4^ CFU/mL, based on the noise levels of the optical setup. The experimental response was measured using 10^6^ CFU/mL of *Bacillus cereus* as model strain, lysed by bacteriophage-coded endolysins PlyB221. The obtained signal matched the expected response, demonstrating the validity of our design and models.

## 1. Introduction

It has been predicted that by 2050, bacteria and their resistance to antibiotics will have more victims than cancer [1]. While new treatments are being developed, detecting threats and implementing interventions to keep resistant pathogenic bacteria from spreading is crucial [2]. Current techniques for bacterial detection and identification are either time-consuming or expensive [3]. Biosensors can overcome these limitations; they are often cheap, fast, specific, sensitive, user-friendly, and sometimes even portable [4]. In this work, we design and fabricate a biosensor for the indirect detection of bacteria. While most bacterial biosensors rely on the specific capture of their target bacteria [5] or their by-products [6], the current research detects the presence of the target bacteria by selectively destroying them. This eliminates the need for functionalization on the sensor surface and reduces the constraints on the fabrication, the storage, and the usage conditions of the biosensor.

When designing a biosensor for bacterial detection, the first question that arises is the nature of the target analyte—i.e., solid, liquid, or gaseous. For our biosensor, a liquid matrix was considered, as this would better fit medical, environmental, and food-related applications. To avoid any influence of the dielectric properties of the aqueous medium on the transducer, an optical detection method was selected. Porous silicon (PSi) is a promising and intensively studied optical transducer [7,8,9,10]. It is characterized by a large internal surface area, rendering it sensitive to minute changes in its environment [7,9]. Its fabrication process is relatively straightforward and can be easily adapted to obtain a wide range of morphologies and properties [9,11]. PSi is characterized by interesting optical properties: it can exhibit either photoluminescence or specific features in its optical reflection spectrum, induced by photonic or interferometric structures. Both of these unique optical characteristics enable an easy and fast read-out of bio-recognition events occurring in the Psi environment [12,13]. Most PSi biosensors are, however, limited by the hindrance of the analyte diffusion inside the porous matrix, which affects their sensitivity [14]. It has been demonstrated, both theoretically and experimentally, that PSi membranes (PSiMs) overcome this challenge by enabling the perfusion of the analyte through the porous matrix, enhancing the sensitivity [15,16,17].

The next step of the biosensor design is the selection of the biological sensing element. The bio-elements of choice for this biosensor are selective lytic enzymes, namely, bacteriophage-encoded endolysins [18,19]. These proteins are able to cleave the bacterial cell wall, inducing bacterial lysis in a targeted manner. Lytic enzymes can be added to the sample volume, eliminating the need for complex functionalization steps and linking the bio-element to the transducer [20].

**Figure 1:**
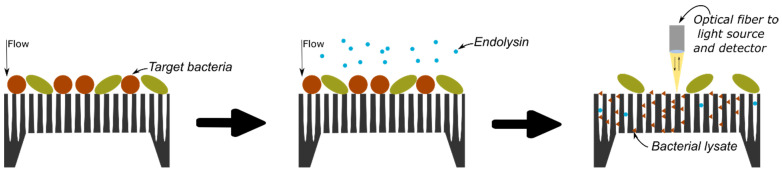
Conceptual protocol for bacterial detection: the analyte is flown through the PSiM and bacteria gather on top of the membrane; a selective endolysin is added and specifically targets one particular bacterium; the resulting bacterial lysate then infiltrates the PSiM, affecting its optical properties.

The protocol of bacterial detection studied in this research is illustrated in Figure 1 and relies on the selective lysis of the target bacteria and the infiltration of the resulting bacterial lysate inside a PSiM. Once bacterial fragments have penetrated the membrane, the PSi optical properties are affected, which can be measured using various optical setups.

This paper focuses on the design of a PSiM-based optical transducer, which is not often documented in the literature. The sensing principle relies on reflective spectroscopy, meaning that the sensor presents a unique pattern of light reflection when interacting with light, which gives insights into the material’s properties. As many PSi nanostructures exhibit these interesting reflection patterns, such as 1D photonic crystals and Fabry–Perot interferometers [10], modeling and simulations were used to select the best-suited geometry for the detection of bacterial lysate. This selection was enabled by the study of the diffusion of bacterial lysate inside a PSiM. The input parameters for the PSi geometry were based on experimental PSi properties. These experimental parameters were analyzed using varying fabrication conditions while ensuring that the bacterial fragments were able to penetrate into the porous layer. The sizes of the bacterial fragments were based on a model of *Bacillus cereus* lysate, as previously studied [21]. Two PSi nanostructures were compared in this diffusion study: (i) a double-layer, relying on reflective interferometric Fourier transform spectroscopy (RIFTS) [11] for the optical read-out, and (ii) a photonic microcavity, relying on the monitoring of its spectral features and/or on its structural color [22]. A schematic illustration of the PSi nanostructures and their spectral features is depicted in Figure S1. The analyte diffusion was modeled using the *Comsol Multiphysics*^®^ v. 5.0 simulation tool.

Once the most suited PSi nanostructure had been selected, the PSiM optical response was also studied in order to optimize the sensor’s sensitivity. The effects of the fabrication parameters on the sensor’s optical response were modeled via transfer matrix simulations [23,24,25,26]. The results of these simulations indicated which were the most appropriate fabrication parameters for our optical sensor. When combining this model with the data collected from the diffusion simulation, a theoretical limit of detection (LOD) could be calculated. This simple analytical model qualifies the impact of key parameters on the sensitivity of the PSi biosensor.

Finally, the modeled sensor was fabricated using a combination of microfabrication techniques, and the protocol of detection was tested using *B. cereus* as the model bacterium and the (bacterio)phage-encoded PlyB221 endolysin as the selective lytic enzyme [27,28].

## 2. Materials and Methods

### 2.1. Model Input Parameters: Characterization of PSi Properties

To build the PSiM models, the properties of PSi were studied experimentally. PSi layer samples were prepared via electrochemical etching of a double-side-polished, boron-doped silicon wafers (<100>, 0.8–0.9 mΩ·cm, 380–400 µm) (Sil’tronix Silicon Technologies, Archamps, France) in HF:ethanol (3:1, *V*/*V*) electrolyte. The PSi samples were first optically characterized using the spectroscopic liquid infiltration method (SLIM), which is based on RIFTS [11]. In short, this method relies on the optical response of the porous layer in the visible light range. The reflectance spectrum of the porous silicon layer is acquired using a 10 mW halogen light source and an Ocean Optics JAZ spectrometer (Ocean Optics, Largo, FL, USA), coupled with an optical fiber. The spectrum is acquired twice, at the exact same spot, using two different filling media. Usually, the first spectrum is acquired in air, and for the second spectrum, the PSi layer is filled with a solvent. Because there is a double reflection on the top and bottom of the PSi layer, the reflectance spectra exhibit Fabry–Pérot fringes, which result from destructive and constructive interferences. When applying a Fourier transform to each spectrum, a frequency peak is observed. The position of each peak translates into the effective optical thickness (EOT), which is related to the layer thickness (*L*) and the average refractive index of the filled porous layer (*n*) such that EOT = 2 *nL*. The EOT values for both filling media are fed into a two-component Bruggeman or Looyenga equation, which enables the computation of both the thickness and porosity of the PSi layer [11].

The PSi samples were also characterized using a Carl Zeiss Ultra 55 SEM (Carl Zeiss, Oberkochen, Germany), both in cross-section (Figure S2) and in top view (Figure S3). The thickness of the PSi layers was verified on the cross-section views. Based on the top views, the pore size distribution could be analyzed using the ImageJ software 1.54h: first, the scale of the image was set and the image was cropped; then, the SEM image was converted into a purely black and white image using fine-tuned threshold values that best reflected the original image. Finally, the “Analyze Particles” feature of the software was used in order to extract the average Feret diameter of the pores and approximate the pore size of the PSi samples.

The sensitivity of the PSi samples was characterized using a variation of the SLIM method. In brief, the reflection spectrum of each PSi sample was measured in different media, namely, air, ethanol, and methanol. The EOT for each medium was recorded and plotted with respect to the refractive index of the medium used. The slope of the linear regression fitted for each sample characterized the sensitivity of the samples.

(1) $$∆EOT_{rel}=\frac{EOT-EOT_{0}}{EOT_{0}}\times 100[\%]$$

### 2.2. PSiM Diffusion Model

The filtration capability of different porous silicon membrane nanostructures was assessed using the *Comsol Multiphysics*^®^ software 5.2, more specifically using the computational fluid dynamics module. The porous silicon membrane was simplified into a 2D problem. Mesoporous silicon is often characterized by branched pores; in this model, pores were assumed to be straight and not interconnected. Only the first few micrometers of the PSiM were modeled, as the optical sensing mostly probed this depth. Two models were constructed, with different geometries: a microcavity and a double-layer. For both models, the filtration of the bacterial lysate was simulated using the creeping flow interface and particle tracing in the fluid flow interface.

In the creeping flow Interface, both the in- and out-flow were pressure-driven. No slip boundary conditions were applied to the walls. The flow was also not impacted by the motion of the particles. The following assumptions were made for the particles: there are no interactions between particles, the particles freeze when hitting the pore walls, and finally, the Stokes drag and gravity are the only forces considered. In the particle tracing interface, two considerations are important: (1) particles do not displace the fluid they occupy and (2) their finite size is not taken into account when modeling particle–wall interactions. Indeed, the particles are treated as point masses. To add the contribution of the particle size to the filtration simulation, two workarounds were added. First, the “wall distance” interface was added to the model in order to calculate the distance from the pore walls; an expression-based boundary condition was then added to all the pore walls, stating that when the distance from the wall is equal or lower than the particle radius, the particle freezes. Secondly, a “fictitious” boundary was added at the top of each porous layer, with an expression-based boundary condition: if the particle diameter is larger than the defined pore size, the particle freezes; if not, the particle can pass through the boundary.

The 2D microcavity model is based on the succession of high- and low-porosity layers, in total 4 stacks of both layers, with a microcavity placed in the middle, as illustrated in Figure S4. The pore size of the high porosity layer was fixed at 40 nm, and the thickness at 100 nm. The low-porosity layer, with pores of 20 nm, was also 100 nm thick. The microcavity, with 40 nm pores, was 200 nm thick. The membrane was 2 µm wide and surrounded by two 1 µm thick fluidic channels, from which the flow entered and exited. All simulation parameters are gathered in Table S2.

The 2D double layer was composed of a 1 µm thick fluidic channel and 20 pores, which represented the first layer of the porous membrane, as depicted in Figure S5. The diameter of the pores was selected using a random Gaussian distribution around 50 ± 25 nm. The second layer of the double PSi layer was simulated as the outlet of the pores, whose diameter was reduced by a factor “diff” that varied from 10 to 40 nm. All simulation parameters are summarized in Table S3.

In both models, the particles had a Gaussian size distribution of 30 ± 20 nm. They were released from a fluidic channel above the PSiMs, and their motion was studied in a time-dependent simulation. The simulated fluid was phosphate-buffered saline (PBS). The flow was pressure-driven: a pressure of 2 bar was applied at the top of the membrane, while the bottom of the membrane remained at atmospheric pressure.

### 2.3. Optical Modeling of a Double-Layered PSiM

The optical response of the multi-layered PSiM was modeled with the transfer matrix method (TMM) using scattering matrices [23,24,25,26]. In this method, the device is presented as a stack of layers of infinite dimensions in the transverse plane. A MATLAB (MathWorks) routine was developed in-house to perform the transfer matrix modeling. First, the reflection of a multi-layered structure was calculated using the TMM with scattering matrices. Then, this reflection spectrum was fed to a RIFTS method algorithm, as detailed in [11].

The simulation was divided into two stages. First, the top layer of the PSiM was simulated as a single layer. Next, the double layer was simulated. The main studied parameters were the porous layers’ thickness and porosity, with the objective of optimizing the sensitivity of the optical sensor. The sensitivity was computed with the relative EOT shifts for different media surrounding the porous layer, using the Looyenga effective media approximation. This approximation was preferred over the Bruggeman one for high-porosity layers [29]. The Looyenga approximation expresses the layer refractive index *n_layer_* in terms of the porosity *P*, the refractive index of the skeleton of the porous material (e.g., Si or SiO_2_) *n_skel_*, and the refractive index of the filling material *n_fill_*:

(2) $$n_{layer}^{2/3}=1-P\cdot n_{skel}^{\frac{2}{3}}+P\cdot n_{fill}^{2/3}$$

This value of *n_layer_* enables the calculation of the relative EOT shifts for different filling media. These EOT values can be plotted with respect to the filling media’s refractive index, fitted using linear regression, and the sensitivity is given by the slope of this regression. 

The range of values used for the layers’ thicknesses, porosities, filling media, and Si skeleton refractive indexes are detailed in Table 1.

### 2.4. Theoretical Limit of Detection

Another MATLAB routine was encoded for the computation of the theoretical LOD. This routine only took into consideration the top layer of the PSiM and was based on the expression of EOT = 2 *nL*. Indeed, upon penetration of the bacterial lysate, the refractive index *n* was affected. The impact on *n* was calculated in terms of the volumetric fraction of bacteria that accumulated inside the porous membrane, following the Gladstone–Dale equation:

(3) $$n-1=\sum_{i}\frac{V_{i}}{V}\left(n_{i}-1\right)$$

In this expression, *V_i_/V* and *n_i_* are the volumetric fraction term and the refractive index of each component of a mixture, respectively. The total volume *V* of both components was calculated in terms of the porosity and size of the porous layer. 

The bacterial lysate was modeled using spherical particles, whose diameters followed a Gaussian distribution around 30 ± 20 nm. The total volume of particles was multiplied by a “*frac*” term, which denoted the fraction of lysate that remained in the porous layer after filtration and was calculated in Section 2.2. The number of particles must then have been related to bacterial concentrations. The bacterial lysate could be modeled by the ribosomes, the proteins, and the DNA and RNA complexes present in the cell, as well as by cell wall fragments. Based on data gathered in the literature, the number of bacterial fragments > 10 nm inside a single bacterial cell could be approximated as 20,000 [30,31,32]. The parameters used for the LOD calculation are detailed in Table 2.

### 2.5. Experimental Validation: Application to the Indirect Detection of Bacteria Via Their Lysis

Based on the simulation results, the PSi biosensor was a Fabry–Perot interferometer, with a double-layered structure. The fabrication of the PSi biosensor is fully detailed elsewhere [21]. The fabrication process started with a 3 in double-etched p++ wafer (<100>, 0.8–0.9 mΩ·cm, 380–400 µm) (Sil’tronix Silicon Technologies). A SiO_2_ and polysilicon layer was deposited and patterned, serving as a mask during the anodization. The mask layout contained 32 dies of 1 cm^2^, with 2 mm openings in the middle, either round or square. The size of the opening was dictated by the spot size of the optical setup. The anodization step began with the removal of the sacrificial layer, followed by the electrochemical etching of the first layer of the PSi sensor at 225 mA/cm^2^ for 30 s. The second layer was etched at 100 mA/cm^2^ for 4090 s in order to obtain ~100 µm thick membranes. The membranes were then opened using dry etching. To assure the stability of the sensor, a thin layer of TiO_2_ was coated inside the PSiM using atomic layer deposition [21].

*B. cereus* ATCC 10987 was used as a reference strain. Bacteria were grown overnight (O/N) in lysogeny broth (LB) or LB-agar plates at 30 °C. In brief, 20 mL of LB was inoculated with 200 µL of bacterial culture and incubated for 3 h at 30 °C. The cultures were then centrifuged at 10,000× *g* for 5 min at room temperature, and the supernatants were resuspended in 20 mL of PBS. This washing step was repeated once, and the optical density (OD_600_) was adjusted to OD_600_ = 0.2 (≈10^6^ CFU/mL). A detailed description of the expression and purification of PlyB221 endolysins can be found elsewhere [27]. The protein concentration was adjusted to 100 µg/mL. 

PSiM samples were integrated in a custom-built polycarbonate fluidic cell. Using a fiber-coupled 10 mW halogen light source and an Ocean Optics JAZ spectrometer, the optical spectra were recorded every 10 s, with a spectral acquisition time of 2 s over a wavelength range of 450–800 nm. Analytes were injected at a flow speed of 1 to 2 µL/min using a Fluigent LINEUP^TM^ fluidic setup (LineUp, Denver, CO, USA). The obtained optical data were analyzed using the RIFTS method in order to obtain the relative EOT.

After pre-wetting the PSiM in PBS for 30 min, a *B. cereus* bacterial suspension was flown through the membrane for 1 h. Next, a PlyB221 endolysin suspension in PBS was added to the fluidic cell for 30 min. The detection was completed by rinsing the PSiM with PBS for 30 min. The relative EOT value was averaged before the introduction of lytic agents over the 40 to 60 min range, as well as after the rinsing step over the 100 to 120 min range. The difference between these average EOT values expresses the relative EOT shift. Negative control tests without bacteria were also carried out. The significance of the relative EOT shift was then established using Student’s *t*-test with a 5% confidence level based on negative control tests in PBS as reference.

## 3. Results

### 3.1. Model Input Parameters: Experimental Characterization of PSi Properties

In order to build the PSiM models, both for diffusion and optical study, the properties of PSi needed to be studied experimentally. For this study, it was decided to use highly doped p-type silicon substrates in order to reach pores size ranging from 10 to 100 nm. With this pore range, most bacterial fragments, as measured in [21], can penetrate into the porous matrix. The possible morphologies of PSi samples were mapped using different fabrication parameters; the main parameter studied here is the current density, as it has been shown that pore dimensions increase with current density [33]. The maximum current density was fixed at 225 mA/cm^2^, as higher values led to highly porous and very fragile layers. It has to be noted that for all samples, the transitional layer was removed via wet etching before the electrochemical etch [11,34].

**Figure 2:**
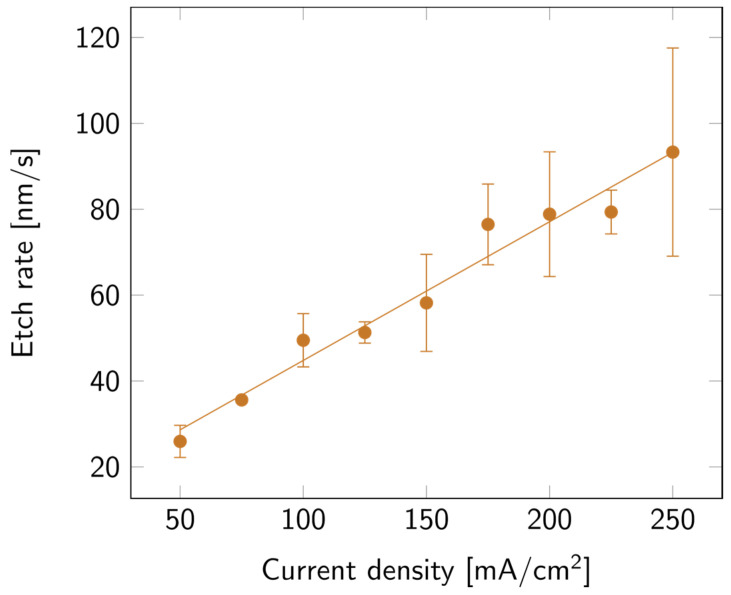
PSi etch rate with varying current densities, on a p++ Si wafer (0.8–0.9 mΩ cm) in HF:EtOH (3:1 *V*/*V*). The error bars represent the deviation between the different measured samples.

SLIM was used to extract the thickness and porosity of the porous layers. The values of the layer thickness were then verified via cross-section SEM measurements. To compare all samples, the collected data were expressed in etch rate instead of thickness. As can be observed in Figure 2, the higher the current density, the faster the etch. This variation appears to be linear with the current density.

**Figure 3:**
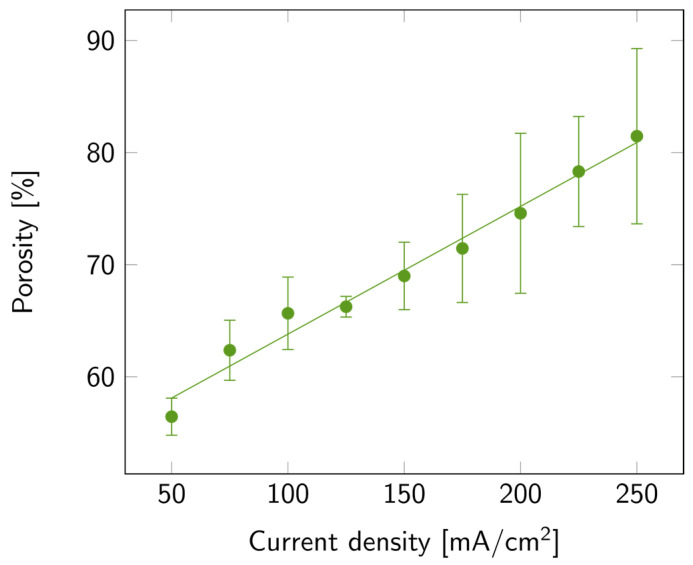
PSi porosity with varying current densities on a p++ Si wafer (0.8–0.9 mΩ cm) in HF:EtOH (3:1 *V*/*V*). The error bars represent the deviation between the different measured samples.

The variation in porosity with current density is illustrated in Figure 3, where a linear increase in porosity with current density is also observed. The maximum average value of porosity was 81%, but samples with up to 90% porosity were measured. The increasing trends of both porosity and etch rate with current density matched the trends previously reported [35]. To explain this trend, one must consider the processes involved in the porosification, among which is the anodic oxide formation and further dissolution in HF-based electrolytes. This process occurs mainly at high current densities [33,35]. Furthermore, the accelerated etch rate may also be linked to the increased rate of silicon anodic dissolution, the enhanced transport of electrolyte to the silicon surface, and the increased concentration of electroactive species [34,35,36].

**Figure 4:**
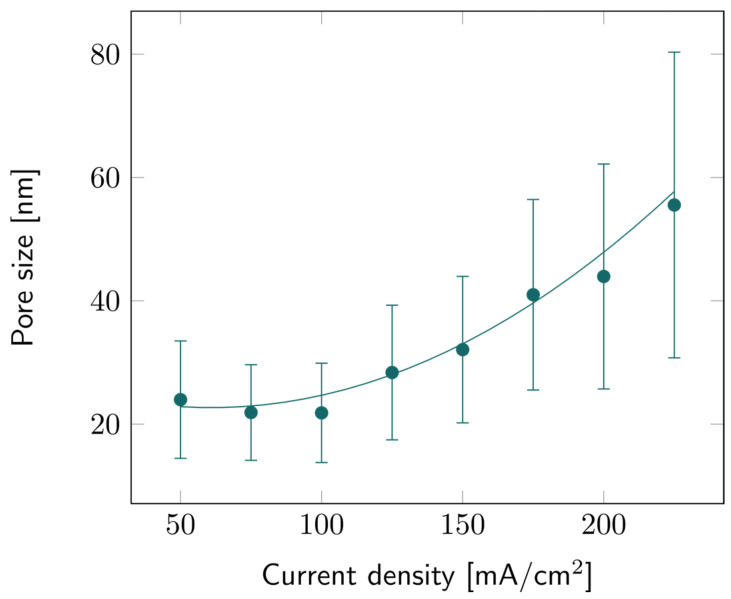
PSi pore size with varying current densities for PSi layers etched on a p++ Si wafer (0.8–0.9 mΩ cm) in HF:EtOH (3:1 *V*/*V*). The error bars represent the deviation in pore size over the measured area, averaged from several samples.

The pore sizes of all PSi samples were characterized based on SEM observations. Image analysis provided data about the average pore size for all current density values, as illustrated in Figure 4. Like porosity and etch rate, the average pore diameter increases with the current density, but the trend is more quadratic than linear. Similar trends have been observed before as well [35]. The large error bars indicate that there is a large pore size distribution, as can also be observed in Figure S3. It can also be noted that the higher the current density, the broader this distribution: while the average pore size for 225 mA/cm^2^ was ~55 nm, larger pores could be observed, up to 80 nm for some samples.

**Figure 5:**
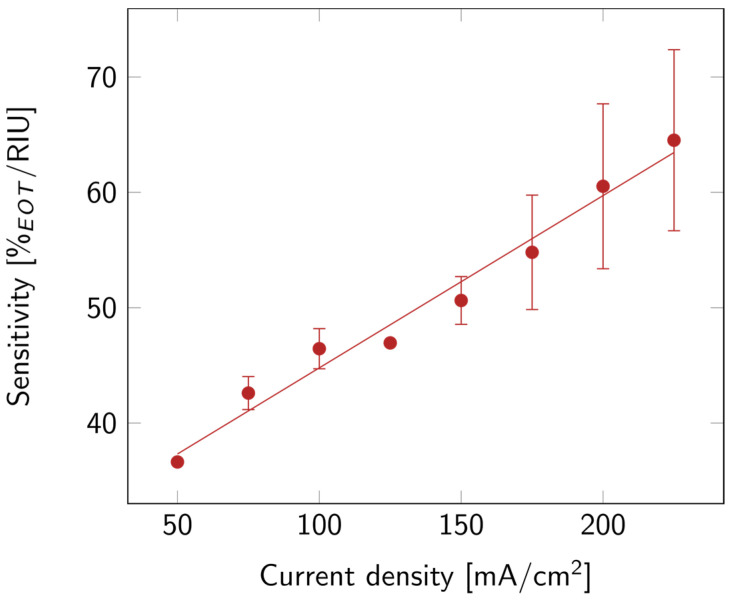
PSi sensitivity with varying current densities for PSi layers etched on a p++ Si wafer (0.8–0.9 mΩ cm) in HF:EtOH (3:1 *V*/*V*). The error bars represent the deviation between the different measured samples.

Finally, the optical sensitivity of the layers was studied. The sensitivity, shown in Figure 5, increased with current density. This might be linked to the increase in porosity. Indeed, the higher the porosity, the more filling material was able to penetrate the layer, and the more it affected the EOT. A summary of all the properties discussed above is available in Table S1.

### 3.2. Modeling the Filtration through PSiMs

The filtration performance of PSiMs was studied using the *Comsol Multiphysics*^®^ software. Two different porous geometries were analyzed: a microcavity and a double layer. The percentage of particles that accumulated inside the porous matrix overtime was studied for both models.

**Figure 6:**
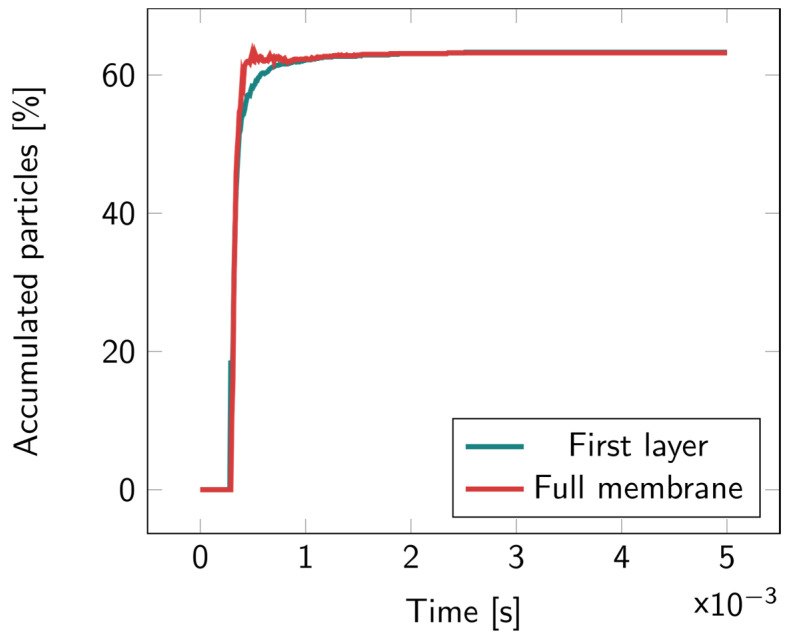
Percentage of the accumulated particles inside the full membrane and inside the first layer of the 2D microcavity over time. The particles were released at the top of the upper fluidic channel at t = 0.

For the 2D microcavity model, particles were counted both in the first layer and in the full membrane. In Figure 6, it can be observed that most of the particles remained in the first layer; no particles were found to accumulate inside the cavity layer. Illustrations of the particles’ motions overtime are available in Figures S6–S8.

**Figure 7:**
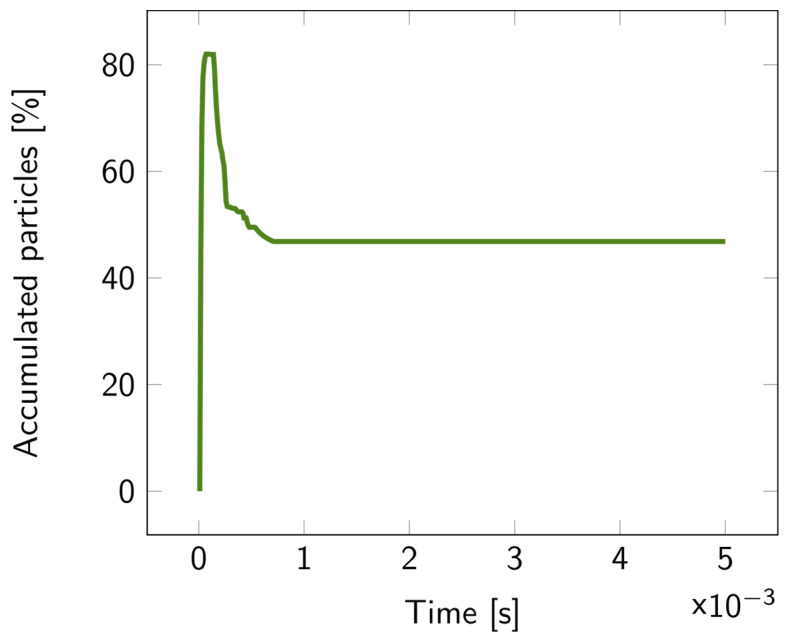
Percentage of the accumulated particles inside the first layer of the 2D double layer overtime, with parameter diff = 20 nm. The particles were released at the top of the upper fluidic channel at t = 0.

**Figure 8:**
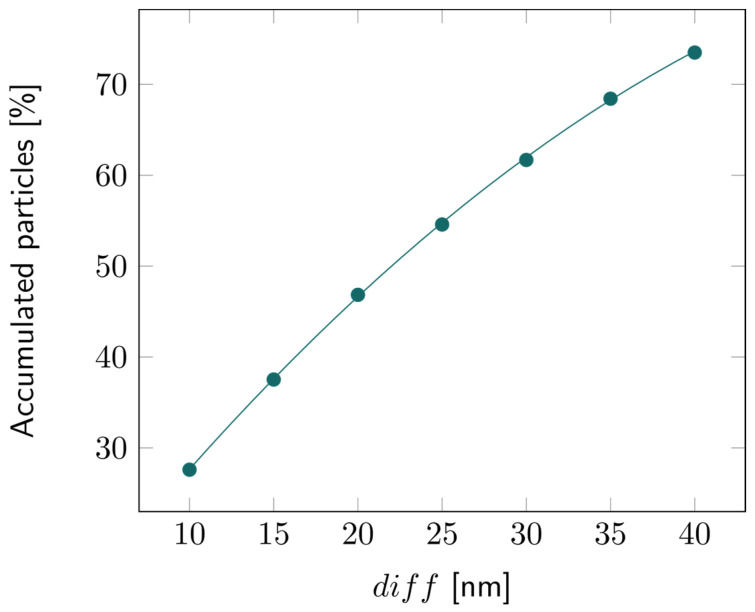
Percentage of the accumulated particles inside the first layer of the 2D double layer with varying diff, that is, with increasing difference in pore diameter between the first and second layers.

For the 2D double-layer model, the particle tracing over time is illustrated in Figures S9–S11. The percentage of accumulated particles in the first layer is depicted in Figure 7. About 50% of the bacterial fragments were retained when the average pore diameter of the second layer was decreased by 20 nm compared to the first layer. This percentage increased even more for larger reductions in pore size, exceeding 70% for a 40 nm difference, as illustrated in Figure 8.

Regardless of the nanostructures, the modeling also brought to light a potential challenge of optical detection: many bacterial fragments do not penetrate inside the porous structure and accumulate on top of it; these might induce optical interferences and pore blockage, which would impact the sensor’s sensitivity.

To select the most suitable nanostructures, one must take into account the optical sensing principle, as illustrated in Figure S1. In the 1D photonic microcavity, the detection is based on the monitoring of the transmission level created by the defect layer within the high-reflection Bragg peak. The spectral position of this transmission level shifts upon the adsorption of analyte in the defects layer. The diffusion model, however, demonstrated that, for bacterial lysate detection, most of the analyte remained in the first layer, and very few bacterial fragments reached the defect layer. The impact on the spectral position of the transmission level was, therefore, minimal, limiting the sensitivity of the transducer, even with a high-resolution optical setup. In the double-layered structure, the optical detection method, called RIFTS, relied on the shift of the effective optical thickness of the first layer, in which most bacterial fragments accumulated. This nanostructure and method of detection was, therefore, more appropriate for the considered application.

### 3.3. Optical Modeling of a Double-Layered PSiM

The optical response of the porous sensors was modeled using TMM simulations. Based on the results of the membrane filtration simulation, microcavities were excluded from the optical response study, as particles were not able to reach the defect layer. Only interferometric multi-layers were, therefore, considered here, and the optical response is based on RIFTS.

**Figure 9:**
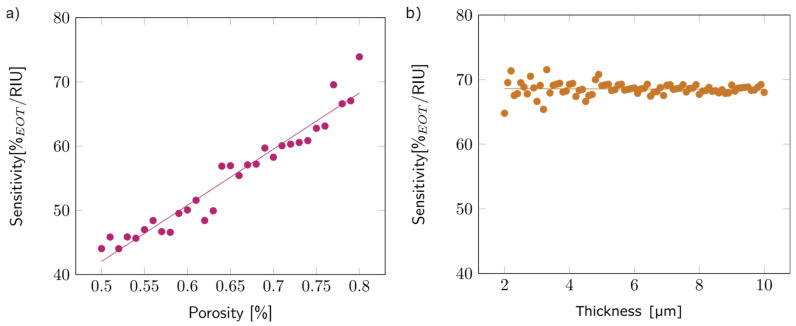
(**a**) Variations in the sensitivity with the porosity of the first layer, with an arbitrary thickness of 5 µm. (**b**) Variations in the sensitivity with the thickness of the first layer, with an arbitrary porosity of 80%.

Once the model was validated (see Figures S12 and S13), the first step of the study concentrated on the first layer, simulated as a single layer surrounded by air. To analyze the effect of porosity on sensitivity, the thickness of the layer was arbitrarily fixed at 5 µm, and the porosity varied between 50 and 80%. Figure 9a depicts the observed variation: sensitivity increased with porosity. This trend can be explained by the higher volume of filling media present inside the porous layer for higher porosities, which increased the overall layer refractive index. The linear variation was similar for other layer thicknesses.

The impact of thickness on sensitivity was studied by varying the layer thickness from 2 to 10 µm while keeping the porosity fixed at 80%. The thickness did not seem to impact the sensitivity (Figure 9b). The same trend was observed for other porosity values.

The second step of the study simulated a double-layered membrane and concentrated on the optimization of the second layer’s porosity. This parameter is important, as light will reflect at the interface between the two layers, but only if there is enough contrast between layers. Indeed, should the refractive index be similar for both layers, then the two layers will be considered as one, and there will be no EOT peak for the first layer.

**Figure 10:**
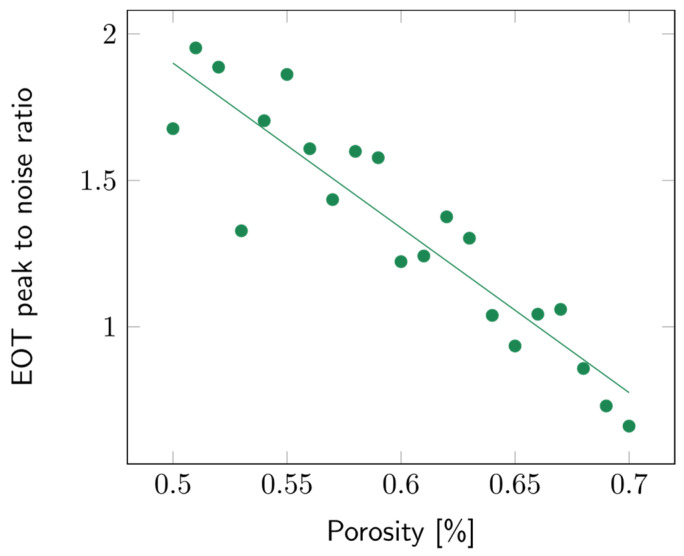
Variations of the peak to noise ratio of the EOT peak with the second layer’s porosity. The filling media’s refractive index was fixed at *n* = 1.3 and the layer thickness at 10 µm.

The impact of the porosity of the second layer was studied over a range from 50 to 70%. The layer thickness was arbitrarily fixed at 10 µm. Variations in porosity did not impact the sensitivity significantly (Figure S14). To study the contrast between the first and second layers, the intensity of the EOT peak was compared to the surrounding noise, which was approximated using the standard deviation of the signal. The higher the EOT peak, the more easily it was picked out and fitted using the RIFTS routine. The peak to noise ratio for a filling medium with refractive index *n* = 1.3 (water), for different contrast layer porosities, is detailed in Figure 10. It can be observed that the lower the second layer’s porosity, the higher the peak to noise ratio.

### 3.4. Theoretical Limit of Detection

**Figure 11:**
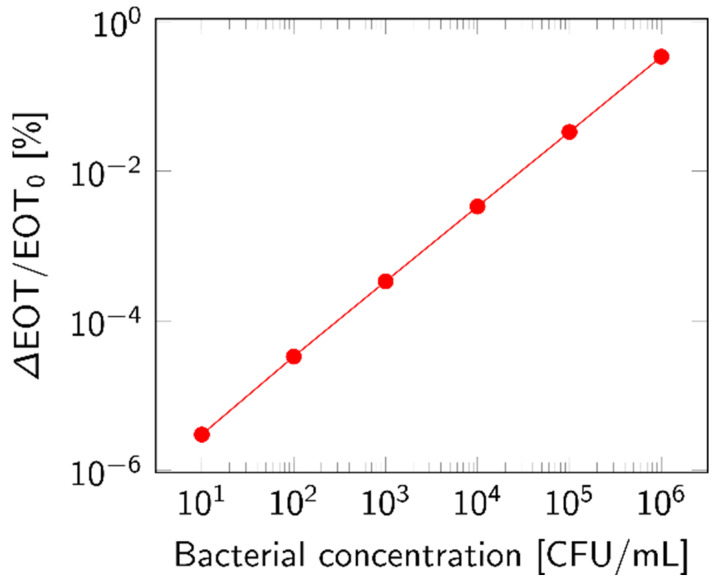
Theoretical response for increasing concentration of bacterial lysate, expressed as relative EOT shift. This response was calculated based on the volumetric fraction of bacterial lysate (*n* = 1.39 RIU) accumulated in the top layer of a PSiM after the percolation of 1 mL of analyte.

The theoretical LOD was approximated by considering only the top layer of the membrane and calculating the EOT shift based on the volumetric fraction of particles accumulated inside the layer. This choice was motivated by the filtration simulations, which found that most of the lysate became trapped in the top layer. The relative EOT shift rose with increasing bacterial concentrations, as plotted in Figure 11. The response was calculated based on 1 mL of analyte. 

To verify these results, a volumetric-based computation was also performed. Water composes 70% of a bacterial cell [30]; based on the remaining 30% of the typical volume of 0.9 µm^3^ for a rod-shaped bacterium [37,38] and on a 0.5 fraction of lysate inside the top layer, we were able to approximate the response with increasing bacterial concentrations. The obtained values were in the same order of magnitude as the values presented in Figure 11.

While a small shift in EOT was observed even for concentrations below 10^3^ CFU/mL, it seems unlikely that these would be distinguished from the baseline signal of the sensor. Indeed, the average error on the relative EOT value of the measurements performed in Section 3.1 was in the 10^−3^ [%] range, which means that the theoretical LOD can be computed as 10^4^ CFU/mL.

The effects of several parameters on the magnitude of the detected shift were also studied. It was observed that the smaller the PSi layer, either in size or in thickness, the higher the EOT shift; indeed, the volume fraction of bacterial lysate was increased and, hence, so was the impact on the EOT. The porosity of the layers and the fraction of lysate inside the layer had similar impacts, but to a lesser extent. By carefully selecting the biosensor design, i.e., preferring a small membrane with a thin first layer and a significant reduction in pore size between layers, the theoretical LOD could be decreased to 10^3^ CFU/mL.

### 3.5. Experimental Validation: Application to the Indirect Detection of Bacteria Via Their Lysis

The results of the different models led to some important design choices:

1. According to the diffusion model, the most suitable PSi nanostructure is a double-layer Fabry–Pérot interferometer, which relies on RIFTS as sensing method;
2. Based on the results of the TMM simulations, the first layer of our double-layered PSi biosensor should be highly porous, while the second layer should have a low porosity;
3. The analytical model for the theoretical detection limit indicated that the first layer of our sensor should be as thin as possible. The dimensions of the membrane should also be as small as possible.

Based on these design parameters, a PSi biosensor was fabricated using a combination of electrochemical etching and standard microfabrication techniques. The full wafer manufacturing process has been used and optimized in several studies [21,28,39], and has been observed to be both robust and repeatable, reaching a success yield of 75%.

**Figure 12:**
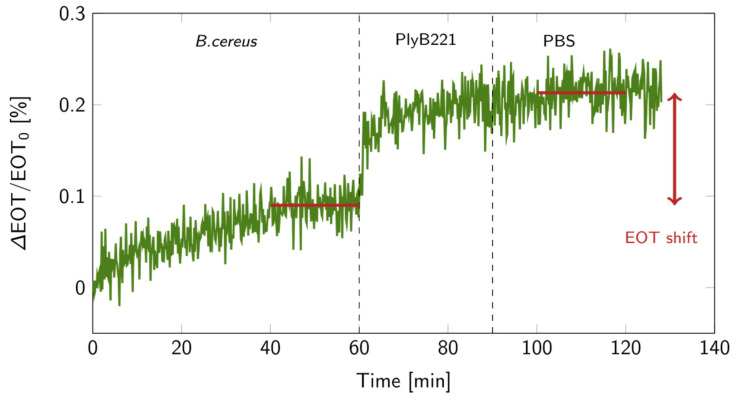
Relative EOT shift over time for a bacterial lysis detection on a PSiM: 1 h in a 10^6^ CFU/mL *B. cereus* suspension, followed by 30 min during which the PlyB221 endolysin was added, and finally a 30 min PBS rinse. The red lines indicate the levels at which the EOT shift was calculated.

**Figure 13:**
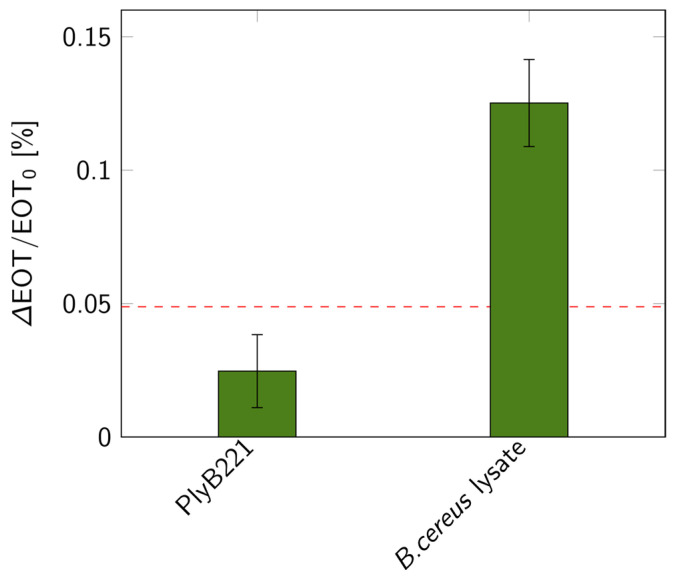
Relative EOT shifts measured on PSiMs for bacterial lysis detections: control test with only PlyB221 endolysin and shift measured when lysing a 10^6^ CFU/mL *B. cereus* suspension (*n* ≥ 3). The dashed red line represents the 3σ level of the signal measured in PBS.

The biosensor’s design was then put to the test for the indirect detection of *B. cereus* through its lysis, following the detection protocol detailed in Figure 1. First, the bacterial suspension was filtered through the PSiM. Then, PlyB221 endolysins were added, causing the bacterial lysate to accumulate inside the PSiM. A rinsing step was finally applied to wash away the endolysin, while the bacterial lysate remained captured inside the porous matrix. The resulting relative EOT shift overtime is illustrated in Figure 12. The relative EOT increased upon the addition of PlyB221 to the fluidic cell. This increase was compared to a negative control, where no bacteria were present, only endolysins. The shift induced by the bacterial detection was significantly higher than this negative control, as shown in Figure 13.

## 4. Discussion

In this work, the design of a PSi-based sensor was studied. This step is not often documented in the PSi community, but is of the upmost importance. The novelty of this work is that it is a comprehensive study of the structure and geometry of a PSi optical transducer, aimed to increase its sensitivity for the detection of bacteria via their lysate.

First, the morphology of PSi layers was studied in order to determine what is possible and which fabrication conditions can be used to achieve the needed porous structure. The PSi sensor must be designed as a filter, enabling the penetration of bacterial fragments. It was observed that the morphology of the porous filter was limited by the fabrication conditions: the highest pore size that could be achieved without risking the mechanical integrity of the sensor was ~55 nm ± 25 nm. With this pore size, a large portion of the bacterial fragments should already be retained. Moreover, the sensitivity to changes in the surrounding refractive index was also the highest with this pore morphology. All these experimental observations were then used as input parameters to build realistic but simplified PSi models.

To study the diffusion of bacterial lysate inside the PSi sensor, the membrane filtration was explored via fluid flow simulations. Two membrane geometries were compared: a microcavity and a double-layer. While the presented 2D models offer only simplified versions of the reality of lysate filtration, the double-layer was observed to be the best membrane geometry for the indirect detection of bacterial lysate, as most fragments were retained in the top porous layer. It was also observed that, in order to optimize the retention of bacterial fragments inside the PSiM top layer, the pore size of the second layer should be as small as possible. It should, however, be large enough to allow the analyte to be pushed through the membrane at a reasonable pressure to avoid any mechanical damage to the sensor. Indeed, the smaller the pores, the higher the pressure needed to push the analyte through at a given flow rate, which is limited by the fragility of the porous membrane. 

While the theoretical fraction of bacterial debris accumulated in the sensor could be quantified, how this value fits the reality remains to be investigated through more complex models or experimental work. Indeed, the nature of the interactions between the lysate fragments and the pore walls is still unknown; electrostatic or other effects might also come into play, but were not considered in the presented models. The effect of the particles on the fluid or the impact of pore blockage, as well as the interaction between bacterial debris, were also not investigated. In addition, the geometry of PSi sensors was also more complex than the presented 2D model: large pores in the top layer may split into two smaller pores for the next layer, and some pores branch out to other pores. A 3D model, based on SEM images of the PSi structure to create the model geometry, may give more accurate results, but would require more computation time and power. Further investigations studying the full membrane, may also enable the study of the fluidic resistance, which affects the flow rate through the membrane and may impact the response. 

The optical response of the sensor was also optimized using transfer matrix simulations. It was observed that the top layer should be as porous as possible. This parameter goes hand in hand with pore size. For the second layer, an optical contrast is needed, which was achieved by reducing the porosity. The TMM was, however, unable to provide sufficient information about the signal intensity and signal-to-noise ratio, as it does not take into account the noise that is present during experimental measurements. This noise can be attributed to, among others, the dark noise, the baseline noise, and the thermal effects of the optical read-out equipment. The noise level is also very important for the determination of the theoretical limit of detection. While it is possible to find specifications of the noise contribution of each component of the optical read-out setup, it remains difficult to translate these in terms of EOT. As an approximation of the noise level, the average error on the EOT fitted using the RIFTS routine was calculated for multiple measurements and averaged. With this approximation and a simple analytical model, it was possible to calculate a theoretical LOD. This LOD was situated in the 10^3^–10^4^ CFU/mL range depending on the PSiM design and remained quite high, but one must keep in mind that it is based on simplified models of the sensor and the analyte. The analytical model also indicated that the thickness of the top layer should be as thin as possible, in order to concentrate the bacterial fragments in the smallest volume possible and hence increase the response.

While the theoretical LOD of 10³ CFU/mL of our sensing platform is not comparable to the sensitivity of current methods such as PCR or bacterial culture, which can both detect as little as one bacterial cell per mL, it is already sufficient for many food safety and even medical applications. This LOD is also within the LOD range of other reflectance-based PSi sensors studied in the literature [40,41,42,43]. A lower LOD can be obtained by switching to other detection methods such as Surface Enhanced Raman Spectroscopy (SERS) or Electrochemical Impedance Spectroscopy (EIS) [6], which have both been combined with PSi transducers [44,45].

Using the key findings of the models and simulations, a PSiM-based biosensor was fabricated using electrochemical etching and standard microfabrication techniques. To verify the design of our sensor, a biosensing experiment was performed for the indirect detection of bacteria via their lysis, using *B. cereus* as model strain. The detection was successful, as the lysis induced a significant shift compared to negative control tests. The EOT shift, amounting to +0.12%, was of the same order of magnitude as the theoretical value predicted for 10^6^ CFU/mL concentrations. The selectivity and versatility of lysis-based detection on PSiMs have already been demonstrated elsewhere [21,28,39], illustrating the added value of combining lytic enzymes and PSi for the development of biosensors. Further investigations have also demonstrated that performing the lysis before the bio-assay, instead of during the bio-assay as was presented in this work, improved the sensitivity of the biosensor: a LOD of 900 CFU/mL was observed for a total assay time of 90 min, and the detection of bacteria in complex media was also demonstrated [21]. This technique, however, requires a control test using the analyte before lysis, which can lengthen the total assay time.

To discuss potential improvements to the sensing platform, one must, however, first discuss the limitations. These limitations concern not only the sensor, but also the measurement equipment. For example, the current optical setup impacts the size of the PSiM; indeed, the area of the porous matrix is limited by the spot size of the optical setup. However, our models suggest that by reducing the size of the PSiM, the LOD can be lowered. By adapting the optical setup, the PSiM can be miniaturized and the sensitivity increased. Moreover, a small PSiM would also improve the mechanical stability of the sensor, which currently limits the flow rate used to push the analyte through the membrane. With improved mechanical stability and a higher flow rate, more bacterial lysate would accumulate inside the porous matrix for the same analyte concentration, enhancing the sensitivity. Another limitation of our current detection platform is the high noise level, which can once again be attributed to both the sensor and the measurement setup. On the sensor’s level, the noise can be linked to surface roughness and thickness variations. These can be minimized, but not fully eliminated. On the level of the optical setup, the noise is intrinsic to the equipment itself and can therefore be improved; for example, the resolution of the spectrometer used in this work was reported as 0.3 to 10 nm, but studies have shown that by upgrading the setup resolution to 0.1 nm, the LOD can be decreased by one order of magnitude [46,47].

Regardless of the limited sensitivity, other hindrances cannot be overcome: while they can be miniaturized at a relatively low cost, the necessity for an optical measurement setup and fluidic integration hinders the development of a PSi-based optical lab-on-chip. Other optical detection methods, such as SERS, are also limited by their even more complex and expensive instrumentation. Ultimately, the choice of detection method is dictated by the specific requirements of the application in terms of sensitivity, cost, and response time. The fabrication of PSi sensors utilizes the affordable electrochemical etching technique and is compatible with the existing silicon microfabrication technology. It could, therefore, be easily and inexpensively mass-produced. For applications that do not require highly sensitive sensors, such as the detection of urinary tract infections or food safety-related detections, our sensing platform could be a versatile, user-friendly, and cost-effective solution.

## 5. Conclusions

This work illustrates the importance of the design of a biosensing platform. While rarely documented, the characterization and modeling of the sensor are crucial to optimizing its sensitivity for a given application. The application illustrated here is the optical detection of bacteria via their lysis. The transducer of choice is a porous silicon membrane. Its morphology, filtration performance, and optical properties have all been studied in depth. This study permitted the selection of the optimal nanostructure for the PSiM, namely, a double-layer structure, and enabled the fine-tuning of the fabrication parameters of the sensor. Using a simplified model of our sensor and sensing experiment, a theoretical response was calculated. This theoretical response was verified experimentally via the detection of *B. cereus* using a phage endolysin to induce the bacterial lysis. The experimental response matched the theoretical one, illustrating the validity of the different models studied in this work.

## Figures and Tables

**Table 1:** Simulation parameters for TMM simulation of the optical response.

Parameter	1st Step	2nd Step
1st layer porosity	50–80 [%]	75 [%]
2nd layer porosity	/	50–65 [%]
1st layer thickness	2–10 [µm]	5 [µm]
2nd layer thickness	/	10 [µm]
Filling media refractive index	1–1.4 [RIU]	
Si skeleton refractive index	3.1 [RIU]	

**Table 2:** Simulation parameters for calculation of the theoretical LOD.

Name	Value	Description
por	80 [%]	1st layer porosity
t	5 [µm]	1st layer thickness
ns	1.7 [RIU]	Si skeleton refractive index
L	1 [mm]	Membrane lateral dimension
nlys	1.3900 [RIU]	Bacterial cytoplasm refractive index
nPBS	1.3348 [RIU]	PBS refractive index
frac	0.5	Fraction of lysate inside the layer
num	20000	Number of particles per bacterial cell
CFU	1–10^6^ [CFU]	Number of bacteria in the analyte in 1 mL

## Data Availability

The data presented in this study are available upon request from the corresponding author.

## Supplementary Materials

The following supporting information can be downloaded at: https://www.mdpi.com/article/10.3390/bios14020104/s1. Figure S1: Example of optical reflectance spectroscopy detection methods for porous silicon transducers: (a) Reflective Interferometric Fourier Spectroscopy (RIFTS) on a porous double-layer, in which the detection is based on a shift of the first layer’s Effective Optical Thickness (EOT) upon analyte adsorption; (b) monitoring of the photonic band gaps of a 1D porous silicon microcavity, whose spectral position is affected by the adsorption of analyte inside the porous matrix. Figure S2: Cross-section SEM images PSi layer etched on a p++ wafer (0.8–0.9 mΩcm) at 200 mA/cm^2^ for 60 s in HF:etOH 3:1 in volume. Figure S3: Top view SEM observations of porous silicon produced with different current densities: 50, 100, 150 and 200 mA/cm^2^. The PSi was etched on a p++ Si wafer (0.8–0.9 mΩcm) in HF:etOH 3:1 in volume. The scale bar represents 100 nm. Figure S4: Schematic cross-section of a microcavity, which was used as geometry for the modelling of membrane filtration. The membrane-based micro-cavity is flanked by two fluidic channels. Figure S5: Schematic cross-section of a double-layer, which was used as geometry for the modelling of membrane filtration. Figure S6: Illustration of the particle tracing inside the 2D microcavity model, just after the release of the particles from the top fluidic channel. The grey gradient depicts the flow rate inside the geometry. Figure S7: Illustration of the particle tracing inside the 2D microcavity model, at an intermediate time, when particles are still flowing through the membrane. The grey gradient depicts the flow rate inside the geometry. Figure S8: Illustration of the particle tracing inside the 2D microcavity model, once all particles have flown through the membrane. The grey gradient depicts the flow rate inside the geometry. Figure S9: Illustration of the particle tracing inside the 2D double layer model, with parameter diff = 20 nm, upon the release of the particles from the top fluidic channel. The grey gradient depicts the flow rate inside the geometry. Figure S10: Illustration of the particle tracing inside the 2D double layer model, with parameter diff = 20 nm, at an intermediate time, when particles are still flowing through the membrane. The grey gradient depicts the flow rate inside the geometry. Figure S11: Illustration of the particle tracing inside the 2D double layer model, with parameter diff = 20 nm, once all particles have flown through the membrane. The grey gradient depicts the flow rate inside the geometry. Figure S12: Interferometric spectra obtained experimentally and theoretically for a single porous layer etched at 200 mA/cm^2^ for 30 s. The values of thickness and porosity used for the simulated spectra were calculated via SLIM on experimentally acquired spectra. Figure S13: FFT of the interferometric spectra obtained experimentally and theoretically for a single porous layer etched at 200 mA/cm^2^ for 30 s. The values of thickness and porosity used for the simulated spectra were calculated via SLIM on experimentally acquired spectra. Figure S14: Variations of the sensitivity with the porosity of the second layer, with an arbitrary thickness of 10 µm. Table S1: Properties of PSi samples etched at different current densities, on a p++ Si wafer (0.8–0.9 mΩ cm) in HF:EtOH (3:1V/V); these values were extracted from SLIM measurements and SEM observations. Table S2: Simulations parameters for the membrane filtration inside a 2D microcavity model. Table S3: Simulations parameters for the membrane filtration inside a 2D microcavity model.
