# Supplementary material for: Design of a Porous Silicon Biosensor: Characterization, Modeling, and Application to the Indirect Detection of Bacteria
Source: Biosensors (Basel). 2024 Feb 17;14(2):104. doi: 10.3390/bios14020104 (PMC10886929; doi:10.3390/bios14020104)
Supplement: Supplementary file 1 [file biosensors-14-00104-s001.zip › biosensors-2801995-supplementary.pdf]

Design of a Porous Silicon Biosensor: Characterization, Modeling, and Application to the Indirect Detection of Bacteria

Roselien Vercauteren [1,†], Clémentine Gevers [1], Jacques Mahillon [2] and Laurent A. Francis [1,*]

[1] Institute of Information and Communication Technologies, Electronics and Applied Mathematics, Université Catholique de Louvain, 1348 Louvain-la-Neuve, Belgium; roselien.vercauteren@vocsens.com (R.V.); clementine.gevers@uclouvain.be (C.G.)
[2] Laboratory of Food and Environmental Microbiology, Earth and Life Institute, Université Catholique de Louvain, 1348 Louvain-la-Neuve, Belgium; jacques.mahillon@uclouvain.be
* Correspondence: laurent.francis@uclouvain.be
† Current address: VOCSens, Rue du Fond Cattelain 1, 1435 Mont-Saint-Guibert, Belgium

1 Introduction

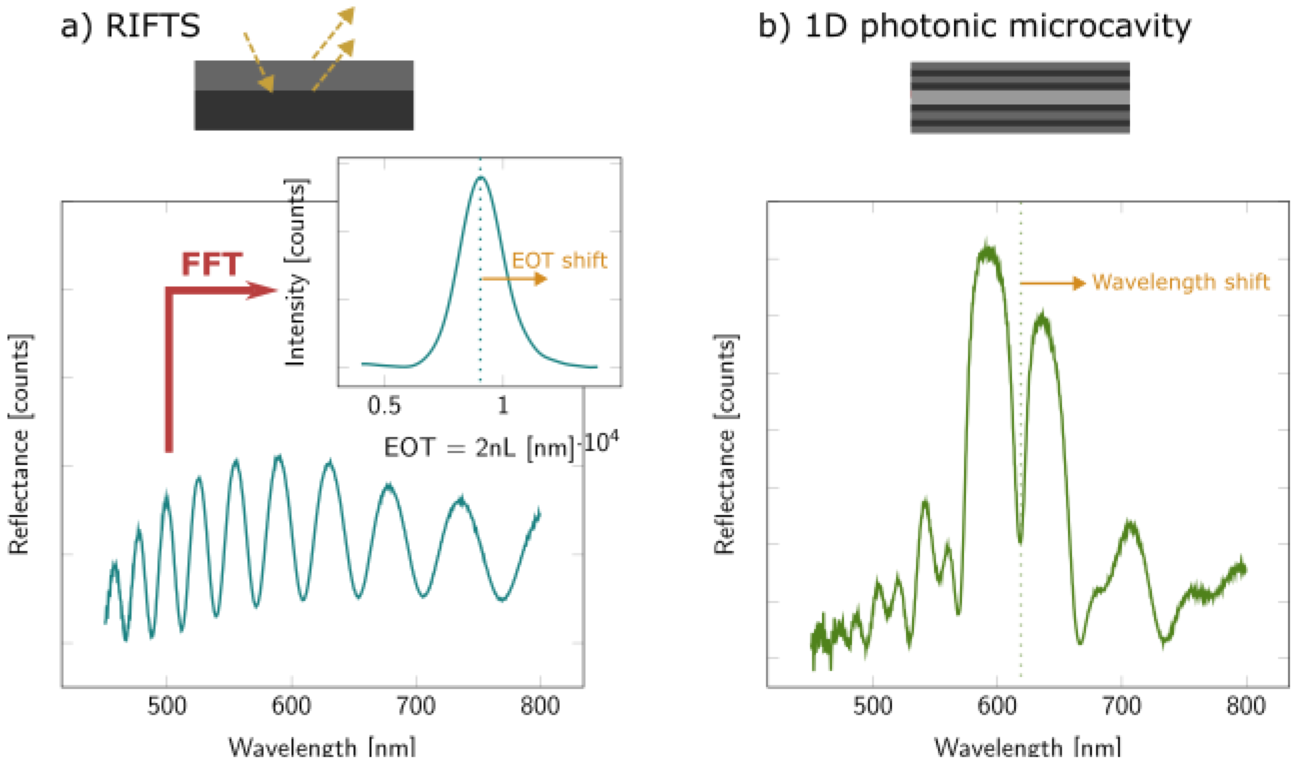

Figure S1. Example of optical reflectance spectroscopy detection methods for porous silicon transducers: (a) Reflective Interferometric Fourier Spectroscopy (RIFTS) on a porous double-layer, in which the detection is based on a shift of the first layer's Effective Optical Thickness (EOT) upon analyte adsorption; (b) monitoring of the photonic band gaps of a 1D porous silicon microcavity, whose spectral position is affected by the adsorption of analyte inside the porous matrix.

2 Porous silicon properties

PSi layer samples were prepared by the electrochemical etching of a double-side polished, boron-doped silicon wafers (<100>, 0.8-0.9 mΩ·cm, 380-400 µm) (Sil'tronix Silicon Technologies). The process was carried out in a custom-made Teflon etch-cell, with a platinum coil as counter-electrode and a potentiostat/galvanostat (PGSTAT302N, Metrohm Belgium) as current source. The porosification was performed in HF:ethanol (3:1, V/V) electrolyte. Aqueous hydrofluoric acid (HF, 49%) and absolute ethanol were obtained from Chem-Lab NV

and from VWR Chemicals, respectively. First, a sacrificial layer was etched at 200 mA/cm2 for 30 sec and then removed with a 2 M aqueous solution of KOH. By etching the sacrificial layer, a larger and more homogeneous pore size is obtained. After a rinsing step, the sample was etched again for 60 sec, at current densities ranging from 50 to 250 mA/cm2.

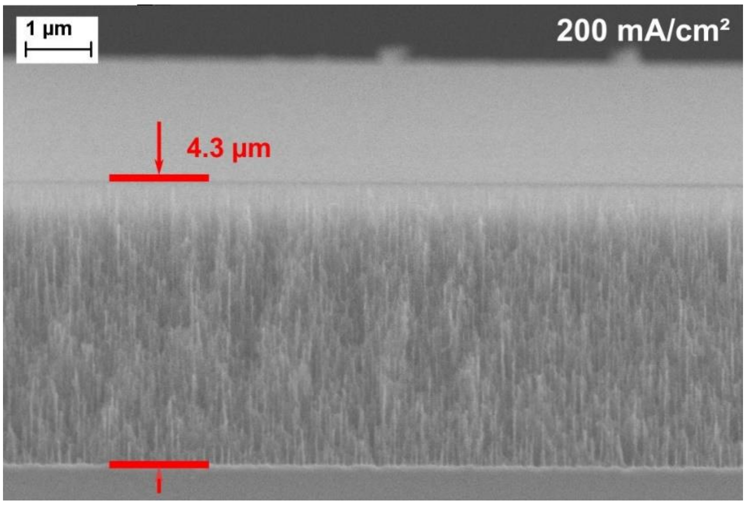

Figure S1. Cross-section SEM images PSi layer etched on a p++ wafer (0.8-0.9 mΩcm) at 200 mA/cm² for 60 s in HF:etOH 3:1 in volume.

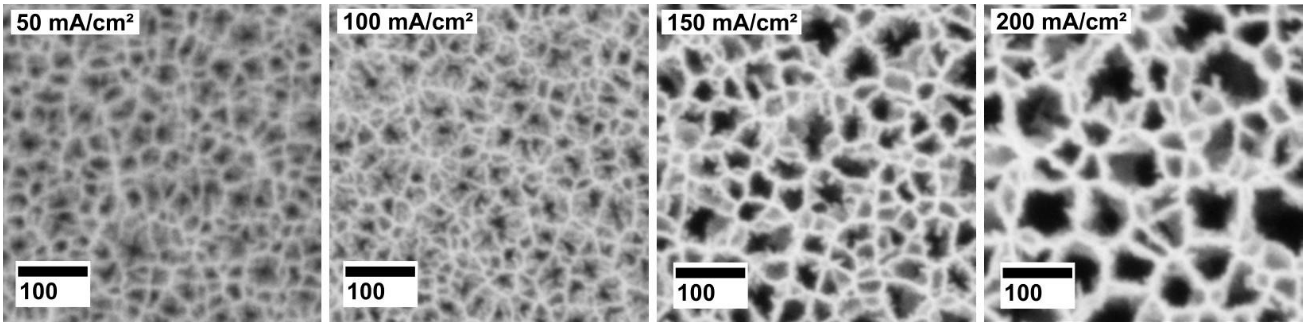

Figure S2. Top view SEM observations of porous silicon produced with different current densities: 50, 100, 150 and 200 mA/cm². The PSi was etched on a p++ Si wafer (0.8-0.9 mΩcm) in HF:etOH 3:1 in volume. The scale bar represents 100 nm.

Current Density [mA/cm²]	Etch rate [nm/s]	Pore size [nm]	Porosity [%]	Sensitivity [$\%_{EOT}$/RIU]
50	25.9 ± 3.73	23.9 ± 9.52	56.4 ± 1.65	36.6 ± 0.03
75	35.6 ± 0.43	21.8 ± 7.75	62.4 ± 2.68	42.6 ± 1.43
100	49.5 ± 6.20	21.8 ± 8.05	65.7 ± 3.23	46.4 ± 1.74
125	51.3 ± 2.47	28.4 ± 10.91	66.2 ± 0.92	46.9 ± 0.18
150	58.2 ± 11.29	32.1 ± 11.88	69.0 ± 3.01	50.6 ± 2.07
175	76.5 ± 9.39	40.9 ± 15.44	71.4 ± 4.82	54.8 ± 4.96
200	78.8 ± 14.51	43.9 ± 18.24	74.6 ± 7.13	60.5 ± 7.15
225	93.3 ± 24.24	55.5 ± 24.78	78.3 ± 7.91	64.5 ± 7.85

Tabel S1. Properties of PSi samples etched at different current densities, on a p++ Si wafer (0.8-0.9 mΩ cm) in HF:EtOH (3:1V/V); these values were extracted from SLIM measurements and SEM observations.

3 PSiM diffusion model

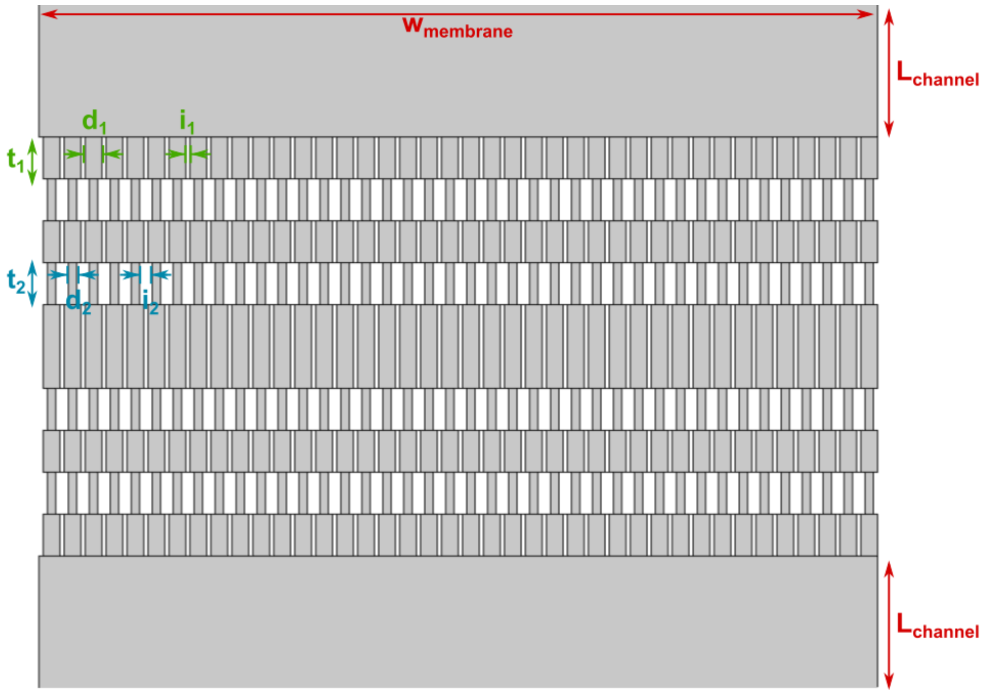

Figure S4. Schematic cross-section of a microcavity, which was used as geometry for the modelling of membrane filtration. The membrane-based micro-cavity is flanked by two fluidic channels.

Name	Value	Description
ρ_{PBS}	1060 [kg/m^3]	Density of PBS
μ_{PBS}	9.04·10^4 [Pa·s]	Dynamic viscosity of PBS Pore
p_1	40 [nm]	diameter of the first layer
p_2	20 [nm]	Pore diameter of the second layer
t_1	100 [nm]	Layer thickness of the first layer
t_2	100 [nm]	Layer thickness of the second layer
i_1	10 [nm]	Inter-pore distance of the first layer
i_2	30 [nm]	Inter-pore distance of the second layer
$w_{membrane}$	2 [μm]	Width of the membrane
$L_{channel}$	1 [μm]	Length of the channel
p_{in}	2 [bar]	Inlet pressure
p_{out}	1 [atm]	Outlet pressure
d_p	30 ± 20 [nm]	Particle diameter
ρ_p	2000 [kg/m³]	Density of the particles

Table S2. Simulations parameters for the membrane filtration inside a 2D microcavity model.

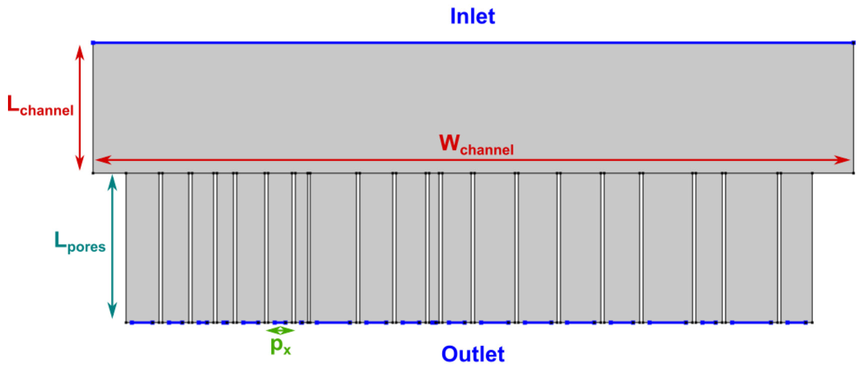

Figure S5. Schematic cross-section of a double-layer, which was used as geometry for the modelling of membrane filtration.

Name	Value	Description
ρ_{PBS}	1060 [kg/m^3]	Density of PBS
μ_{PBS}	9.04e ´4 [Pa¨s]	Dynamic viscosity of PBS Pore
p_x	50 ± 25 [nm]	diameter of the first layer
diff	10-40 [nm]	Difference in pore size between layers
i	5 [nm]	Inter-pore distance of the first layer
L_{pores}	2.3 [μm]	Length of the pores
$w_{channel}$	1.1 [μm]	Width of the channel
$L_{channel}$	200 [nm]	Length of the channel
p_{in}	2 [bar]	Inlet pressure
p_{out}	1 [atm]	Outlet pressure
d_p	30 ± 20 [nm]	Particle diameter
ρ_p	2000 [kg/m³]	Density of the particles

Table S3. Simulations parameters for the membrane filtration inside a 2D microcavity model.

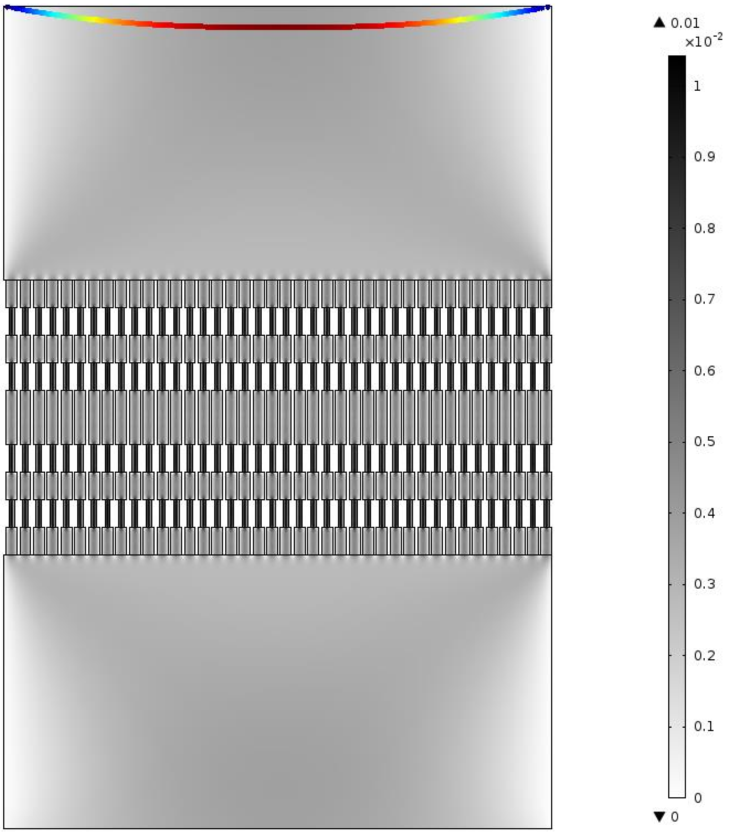

Figure S6. Illustration of the particle tracing inside the 2D microcavity model, just after the release of the particles from the top fluidic channel. The grey gradient depicts the flow rate inside the geometry.

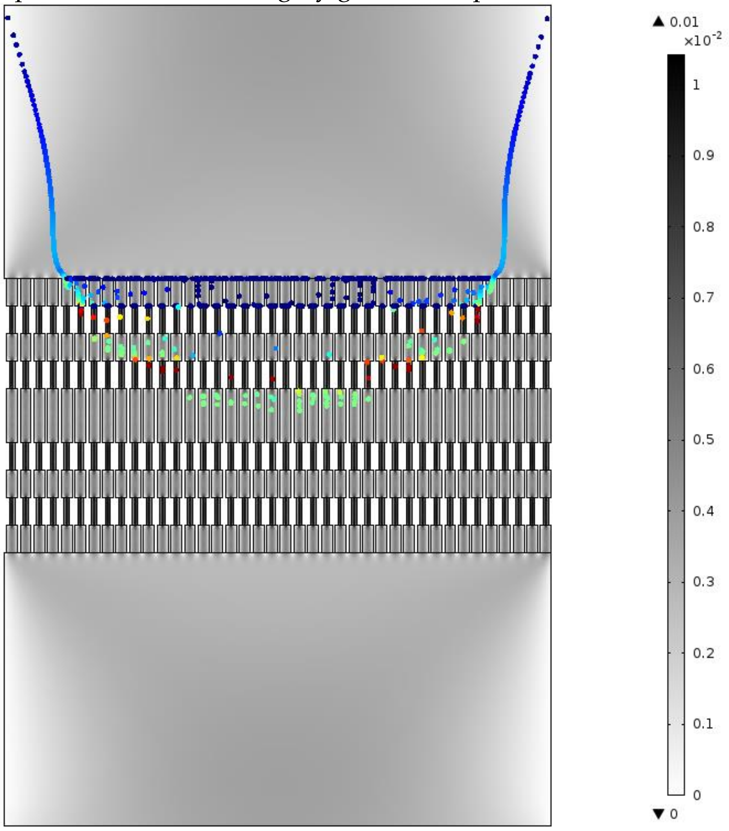

Figure S7. Illustration of the particle tracing inside the 2D microcavity model, at an intermediate time, when particles are still flowing through the membrane. The grey gradient depicts the flow rate inside the geometry.

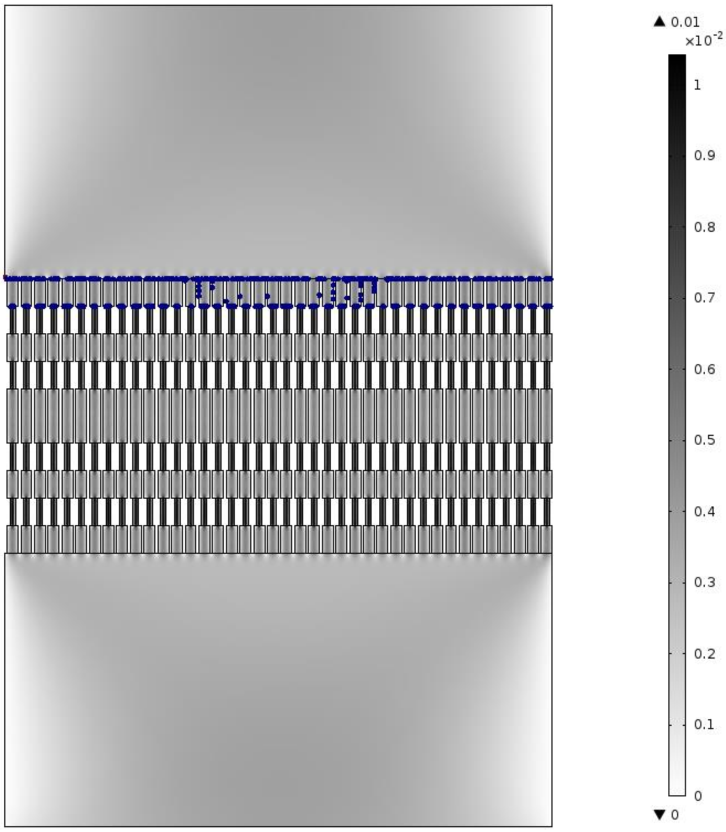

Figure S8. Illustration of the particle tracing inside the 2D microcavity model, once all particles have flown through the membrane. The grey gradient depicts the flow rate inside the geometry.

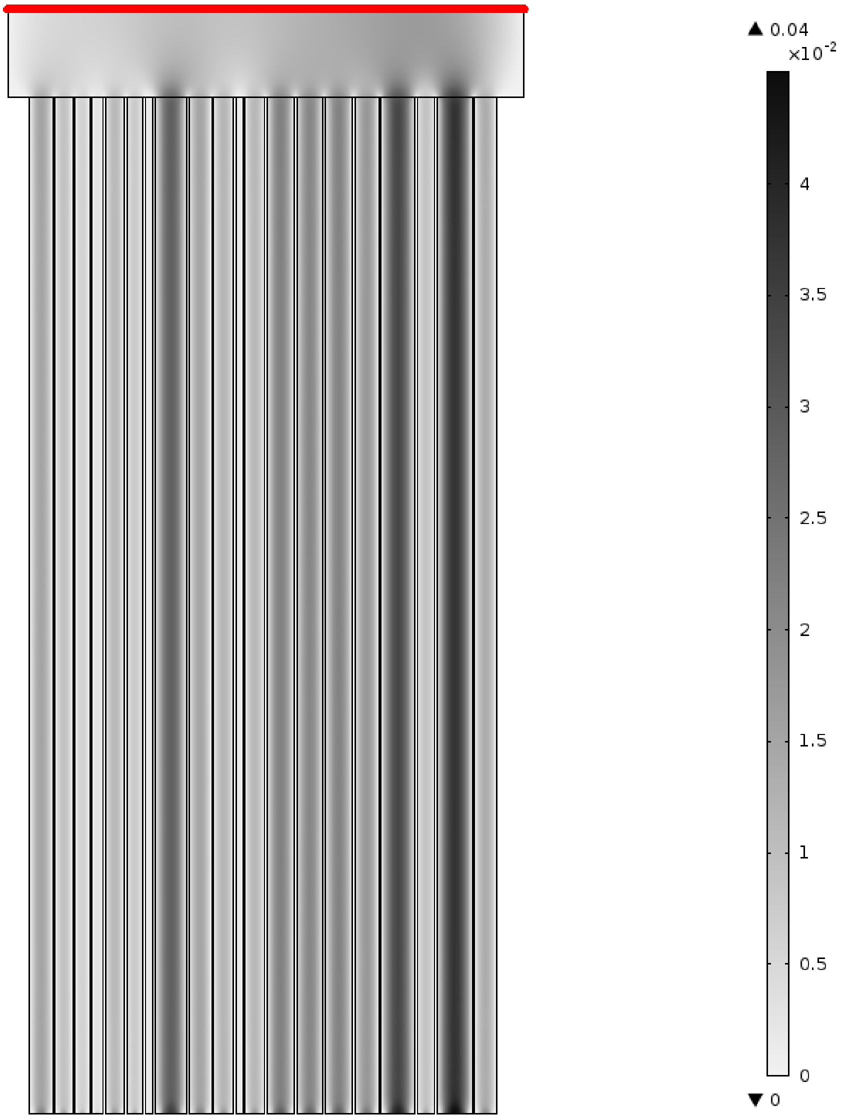

Figure S9. Illustration of the particle tracing inside the 2D double layer model, with parameter diff=20 nm, upon the release of the particles from the top fluidic channel. The grey gradient depicts the flow rate inside the geometry.

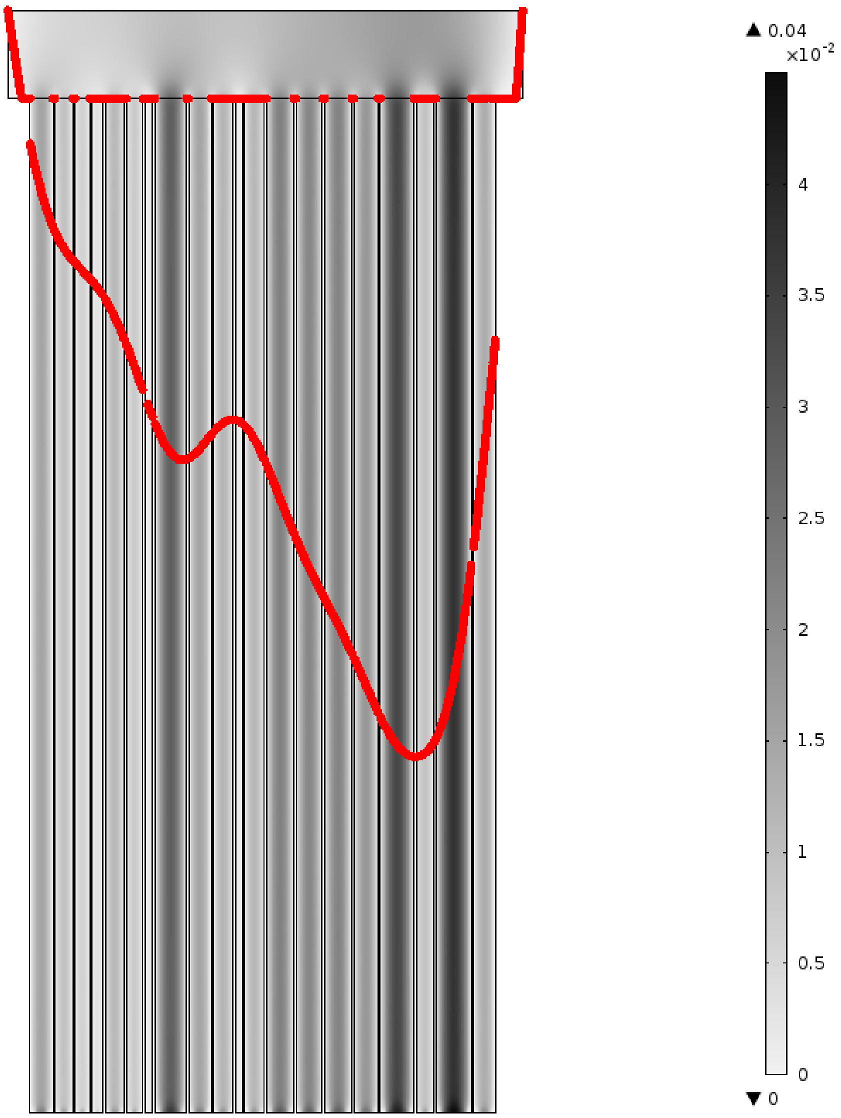

Figure S10. Illustration of the particle tracing inside the 2D double layer model, with parameter diff=20 nm, at an intermediate time, when particles are still flowing through the membrane. The grey gradient depicts the flow rate inside the geometry.

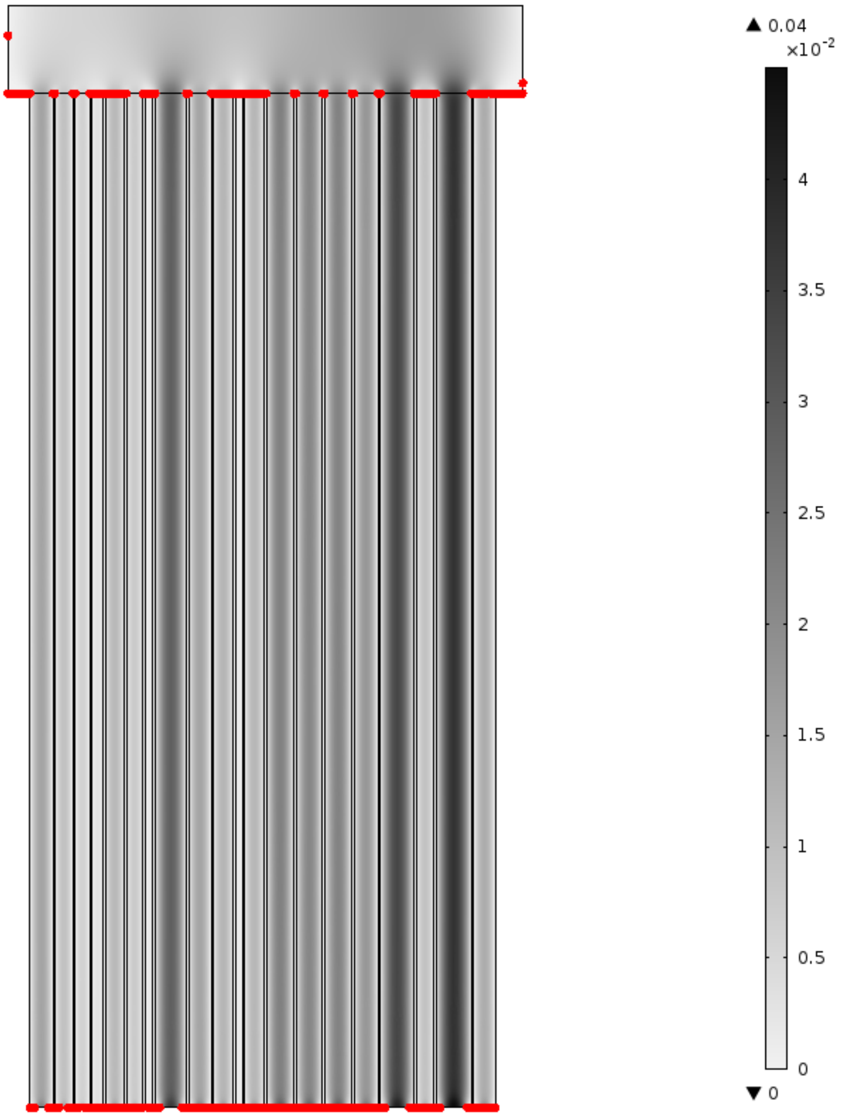

Figure S11. Illustration of the particle tracing inside the 2D double layer model, with parameter diff=20 nm, once all particles have flown through the membrane. The grey gradient depicts the flow rate inside the geometry.

4 Optical modelling of a double-layered PSi membrane

Model validation

Before diving into the optical response simulation, the model is validated by comparing the simulated theoretical optical output to an experimentally measured one. The experimental sample is a single porous layer etched at 200 mA/cm^2 for 30 sec. The thickness and porosity of the sample are measured using the SLIM method: the thickness is 3.3 µm and the porosity is 75%. These parameters are used in the TMM model in order to calculate the optical signal. Both experimental and simulation optical spectra are depicted in **Figure S**: a fringe pattern can be observed in both spectra, but it remains difficult to establish if the frequency of the fringes is similar.

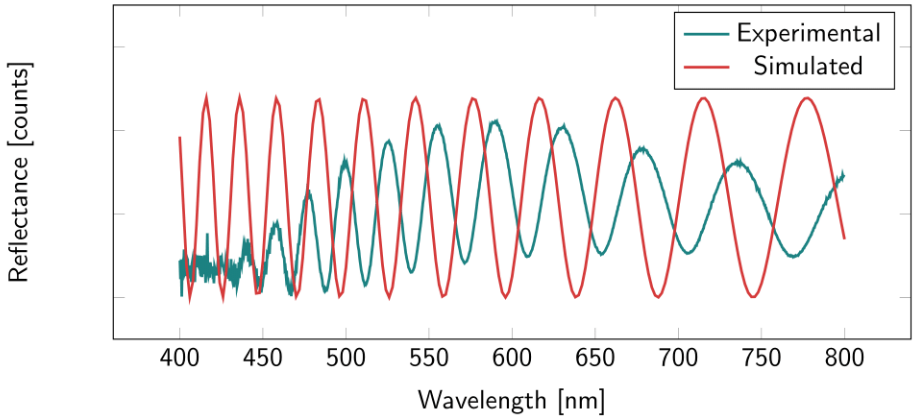

Figure S12. Interferometric spectra obtained experimentally and theoretically for a single porous layer etched at 200 mA/cm² for 30s. The values of thickness and porosity used for the simulated spectra were calculated via SLIM on experimentally acquired spectra.

A RIFTS routine is applied to both optical spectra in order to comparethe fringe frequency, which is reflected by the EOT value. The resulting FFT is illustrated in **Figure S**, where one can see that both spectra are centered around the same $2nL$ values, namely 9000 nm. More precisely, the EOT value of the experimental spectra is 9017 nm, and the simulated one is 8965 nm. The difference between these two values remains minor and can be attributed to the fact that the porosity and thickness used for the simulation are fitted with SLIM and not exact measurements. Moreover, the transfer matric model considers an ideal situation, with no losses, absorption or wavelength dispersion, which also may explain the slight deviation between theory and experiment. It can however be concluded that the TMM simulation is able to model porous silicon layers.

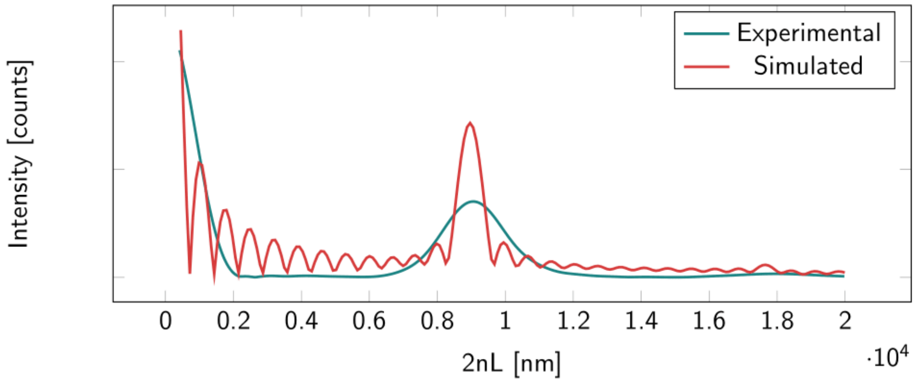

Figure S13. FFT of the interferometric spectra obtained experimentally and theoretically for a single porous layer etched at 200 mA/cm² for 30s. The values of thickness and porosity used for the simulated spectra were calculated via SLIM on experimentally acquired spectra.

4.1 Results

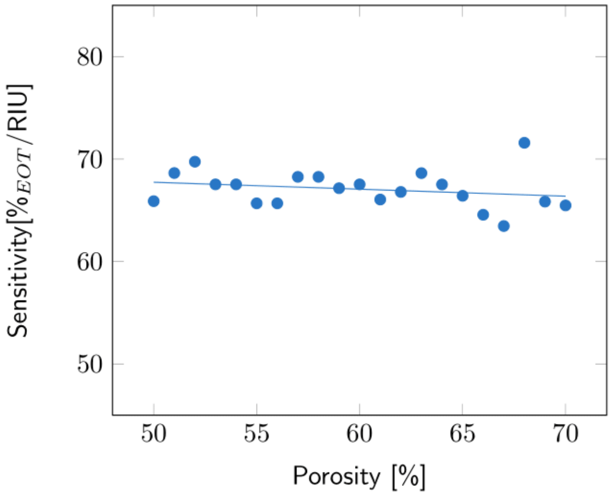

Figure S14. Variations of the sensitivity with the porosity of the second layer, with an arbitrary thickness of 10 µm.
